# Application of negative tissue interstitial pressure improves functional capillary density after hemorrhagic shock in the absence of volume resuscitation

**DOI:** 10.14814/phy2.14783

**Published:** 2021-03-04

**Authors:** Vinay P. Jani, Vivek P. Jani, Carlos J. Munoz, Krianthan Govender, Alexander T. Williams, Pedro Cabrales

**Affiliations:** ^1^ Department of Bioengineering University of California San Diego La Jolla CA USA; ^2^ Division of Cardiology Department of Medicine The Johns Hopkins University The Johns Hopkins School of Medicine Baltimore MD USA

**Keywords:** functional capillary density, microcirculation, negative pressure, Starling forces

## Abstract

Microvascular fluid exchange is primarily dependent on Starling forces and both the active and passive myogenic response of arterioles and post‐capillary venules. Arterioles are classically considered resistance vessels, while venules are considered capacitance vessels with high distensibility and low tonic sympathetic stimulation at rest. However, few studies have investigated the effects of modulating interstitial hydrostatic pressure, particularly in the context of hemorrhagic shock. The objective of this study was to investigate the mechanics of arterioles and functional capillary density (FCD) during application of negative tissue interstitial pressure after 40% total blood volume hemorrhagic shock. In this study, we characterized systemic and microcirculatory hemodynamic parameters, including FCD, in hamsters instrumented with a dorsal window chamber and a custom‐designed negative pressure application device via intravital microscopy. In large arterioles, application of negative pressure after hemorrhagic shock resulted in a 13 ± 11% decrease in flow compared with only a 7 ± 9% decrease in flow after hemorrhagic shock alone after 90 minutes. In post‐capillary venules, however, application of negative pressure after hemorrhagic shock resulted in a 31 ± 4% decrease in flow compared with only an 8 ± 5% decrease in flow after hemorrhagic shock alone after 90 minutes. Normalized FCD was observed to significantly improve after application of negative pressure after hemorrhagic shock (0.66 ± 0.02) compared to hemorrhagic shock without application of negative pressure (0.50 ± 0.04). Our study demonstrates that application of negative pressure acutely improves FCD during hemorrhagic shock, though it does not normalize FCD. These results suggest that by increasing the hydrostatic pressure gradient between the microvasculature and interstitium, microvascular perfusion can be transiently restored in the absence of volume resuscitation. This study has significant clinical implications, particularly in negative pressure wound therapy, and offers an alternative mechanism to improve microvascular perfusion during hypovolemic shock.

## Graphical abstract

The objective of this study was to investigate the mechanics of arterioles and functional capillary density (FCD) during application of negative tissue interstitial pressure after 40% total blood volume hemorrhagic shock. Normalized FCD was observed to significantly improve after application of negative pressure after hemorrhagic shock (0.66 ± 0.02) compared to hemorrhagic shock without application of negative pressure (0.50 ± 0.04). Our study demonstrates that application of negative pressure acutely improves FCD during hemorrhagic shock, though it does not normalize FCD.

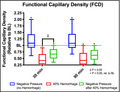

## 1 INTRODUCTION

Severe hemorrhagic shock is a known cause of widespread organ failure even after correction of systemic hemodynamics with transfusion(Cabrales et al., ,^2004^, ^2007^). Despite correction of systemic hemodynamics, microvascular perfusion often remains impaired(Cabrales et al., ,^2004^, ^2007^). *In vivo*, microvascular fluid exchange is primarily dependent on Starling forces. Microvascular fluid flux is a function of capillary and interstitial hydrostatic and oncotic pressures. The volumetric filtration flux of fluid through the capillary wall per unit area, $J_{V}/A$, is described by the Starling equation, shown below,

(1) $$J_{V}/A=L_{P}\left[\left(P_{c}‐P_{i}\right)‐\sigma \left(\pi_{c}‐\pi_{i}\right)\right]$$

where $P_{c}$ and $P_{i}$ are the capillary and interstitial hydrostatic pressures, respectively, $\pi_{c}$ and $\pi_{i}$ are the capillary and interstitial oncotic pressures, respectively, $L_{P}$ is the permeability of the capillary wall, and σ is Staverman's oncotic reflection coefficient(Hu et al., 2000; Levick & Michel, 2010). Under normal physiological conditions, oncotic pressure drives vascular resorption as the vascular hydrostatic pressure must be greater than the interstitial hydrostatic pressure to reduce vascular compression(Effros & Parker, 2009). Recent evidence suggests that the endothelial glycocalyx, a complex layer of glycoproteins and proteoglycans, plays a larger role in microvascular fluid absorption and has led to a revision of Starling's principles. The large porous structure of the glycocalyx creates a “protected region” proximal to the endothelial cell surface, which possesses a low oncotic pressure relative to the protein‐rich glycocalyx, enhancing volumetric filtration(Becker et al., 2010). Modifications to the glycocalyx in diabetes, shock, and atherosclerosis consequently have unexpected effects on the microvascular fluid flux that were previously underappreciated. In these cases, interstitial edema can form due to changes in hydrostatic pressure, as observed in pulmonary edema secondary to left ventricular failure(Murray, 2011), or an increase in vascular permeability and subsequent loss of the glycocalyx‐endothelial oncotic pressure gradient, as observed in acute respiratory distress syndrome (ARDS)(Monnet et al., 2007; Murray, 2011). An understanding of the Starling forces can be leveraged to reverse interstitial edema. For instance, it has been shown that interstitial edema can be reversed by increasing plasma oncotic pressure(Effros & Parker, 2009). However, few studies have investigated the effects of modulating interstitial hydrostatic pressure, particularly in the context of hemorrhagic shock.

In addition to Starling forces, microvascular fluid flux is dependent on both the active and passive myogenic properties of arterioles and post‐capillary venules. Arterioles are classically considered resistance vessels, with tonic sympathetic stimulation predominantly determining resting wall tension(Bohlen and Lash, 1994;23(6_pt_^1^):^757^–^764^.; Lash & Bohlen, 1991; Lash et al., 1991). The active myogenic response is a calcium‐dependent process and is triggered by increased wall tension or vascular stretch, generally secondary to vessel distension from increased hydrostatic pressure(Johnson, 1991). The passive elastic properties result from the connective tissue wall composition: elastin conferring elasticity and collagen conferring stiffness. Importantly, alterations in passive stiffness adversely affect pressure sensing and can inhibit the myogenic response, as observed in microvascular angiopathy secondary to diabetes and hypertension(Bohlen and Lash, 1994;23(6_pt_^1^):^757^–^764^.; Hill & Ege, 1994). Arteriolar tone also controls FCD and thus is responsible for modulating microvascular perfusion(Honig et al., 1980, 1982; Tyml & Groom, 1980). The passive circumferential properties of venules are Hookean at any given level of sympathetic tone due to their thin walls(Lang & Johns, 1987; Shoukas & Bohlen, 1990). Few studies, however, have investigated the effects of changing tissue interstitial pressure on microvascular myogenic properties.

Methods to correct impaired perfusion during hemorrhage have centered around altering blood rheology, modulating Starling forces, and by pharmacologically increasing sympathetic stimulation of the microvasculature. The viscosity of blood in circulation has shown to be an important factor in resuscitation from hemorrhagic shock. Many studies have established that loss of blood viscosity from hemodilution and transfusion result in microvascular endothelial injury, tissue damage, and death(Cabrales et al., ,^2004^, ^2007^; Kerger et al., 1999). As a result, it has been hypothesized that supplemental transfusion with high viscosity plasma expanders may improve perfusion. Much work has demonstrated that high viscosity plasma expanders used concomitantly with blood transfusion improve microvascular perfusion and FCD during hemorrhagic shock(Cabrales et al., ,^2004^, ^2007^; Kerger et al., 1999). However, these methods may result in extreme hemodilution, which results in decreased O_2_ carrying capacity and impaired FCD(Cabrales et al., ,^2004^, ^2007^; Kerger et al., 1999). We hypothesize that application of a negative tissue interstitial pressure may serve to improve microvascular perfusion during hemorrhagic shock to compensate for the loss in hydrostatic pressure gradient, and as such could act as an alternative to fluid resuscitation. Therefore, the objective of this study was to investigate the mechanics of dynamics in arterioles and FCD during application of negative tissue interstitial pressure after hemorrhagic shock.

## 2 MATERIALS AND METHODS

### 2.1 Animal preparation

Male Syrian Golden hamsters weighing 55 to 70 g (Charles River Laboratories, Boston, MA) were fitted with a dorsal skinfold window chamber model. Care and handling of hamsters abided by the NIH Guide for the Care and Use of Laboratory Animals. Approval of the study was given by the University of California San Diego Institutional Animal Care and Use Committee. The window chamber preparation is a well‐established model of microvascular blood flow in intact tissue that can be studied without anesthesia(Endrich et al., 1980). Tissues studied in this model include skeletal muscle and subcutaneous connective tissue, described in detail elsewhere(Endrich et al., 1980). Chamber implantation was performed under anesthesia (200 mg/kg ketamine and 10 mg/kg xylazine IP). Briefly, the chamber consists of 2 titanium frames with a 15‐mm‐diameter circulation observation window; 1 frame of the chamber was placed in direct contact with animal skin. Sutures were placed to lift the dorsal skin away from the animal. The skinfold was removed following the outline of the window until only a thin monolayer of muscle and skin remained. A cover glass was placed on the exposed tissue and held in place by the other frame under a drop of saline. A minimum of 48 hours was allowed for recovery prior to catheterization. Chambers with signs of edema, bleeding, or neovascularization were not used. Animals were re‐anesthetized as described, and carotid (arterial) and jugular (venous) catheters were implanted and exteriorized at the dorsal side of the neck, where they were attached to the chamber frame for easy access. The animals were then allowed another day for recovery prior to experimentation. Experimentation was performed in the awake, unanesthetized state.

### 2.2 Inclusion criteria

Inclusion criteria included the following: (1) systemic parameters within reference range at baseline (heart rate (HR) >340 beats per minute, mean arterial pressure (MAP) >80 mmHg and <130 mmHg, systemic hematocrit (Hct) >45%, PaO_2_ > 50 mmHg), and (2) no signs of edema, bleeding, or unusual neovascularization of tissue in the chamber under 20x magnification. Note that PaO_2_ > 50 mmHg is considered normal for hamsters; however, microvascular PO_2_ distribution is similar to that of other rodents(Cabrales et al., 2005).

### 2.3 Negative tissue interstitial pressure application protocol

**FIGURE 1:**
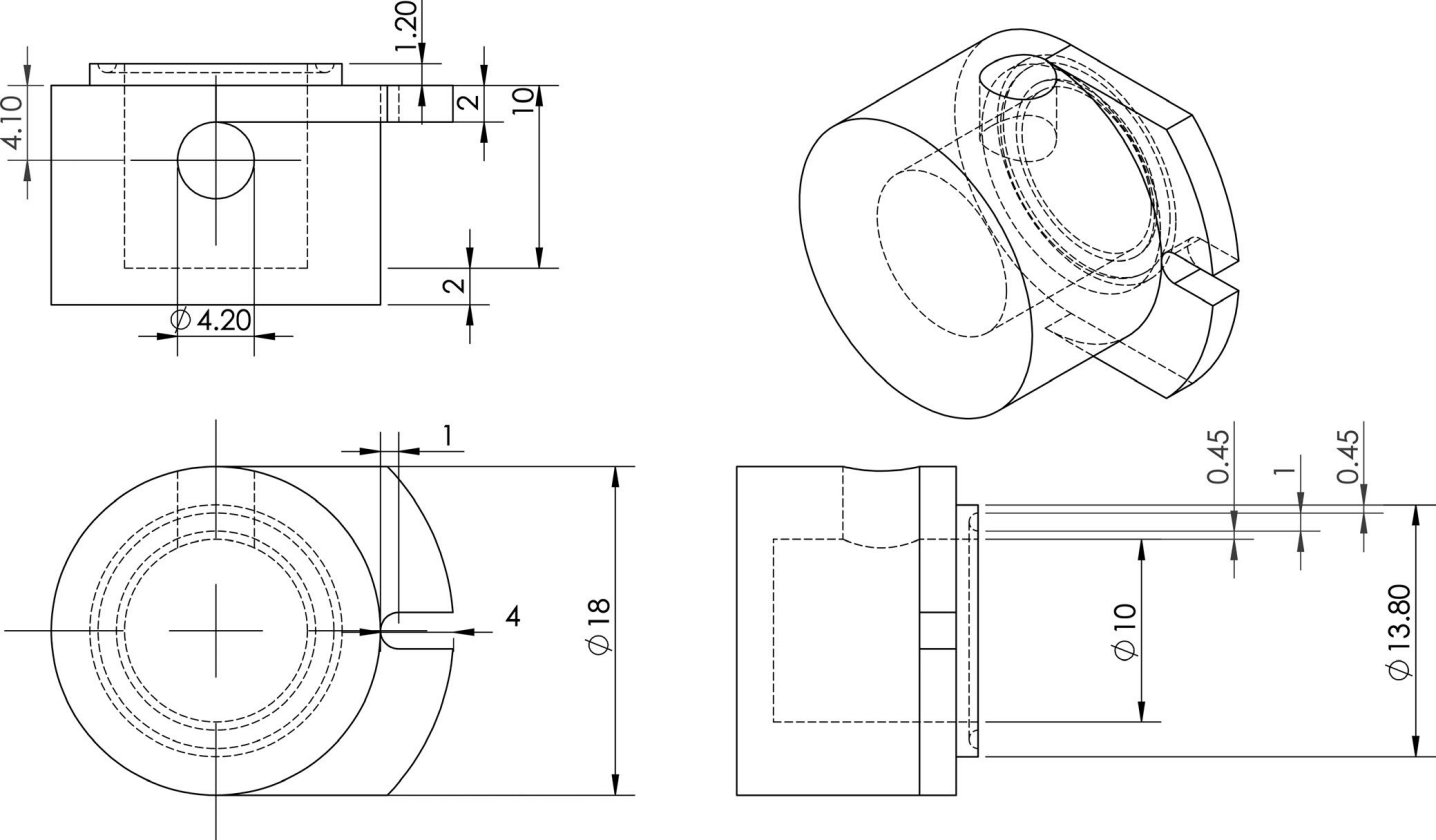
Schematic of Custom‐designed Negative Pressure Chamber. A negative pressure chamber was designed using SolidWorks and constructed using acrylic in order to locally apply negative pressure to the hamster dorsal skin fold window chamber model.

A 15‐mm‐diameter circular chamber was constructed from acrylic for application of negative tissue interstitial pressure. Device specifications are shown in Figure 1. Application of negative tissue interstitial pressure involved attachment of the device to the intact subcutaneous skin on the back of the chamber. Briefly, a copious amount of high‐grade silicon grease (Anchor Electronics, Santa Clara, CA) was applied to the 1.2 mm lip of the device with a wooden applicator and placed in direct contact with the subcutaneous skin and secured onto the chamber. To create a vacuum seal, a luer adapter was fixed to the port located on the side of the chamber and connected to a clear PVC 1/8‐inch inner diameter tubing (Fisher Scientific, Waltham, MA). The tubing was secured to a 10‐mL syringe and pressure transducer (MP150; Biopac Systems, Santa Barbara, CA) with a three‐way luer adapter. Pressure was adjusted by modulating the volume of saline in the 10 mL syringe. The experimental setup is described in detail elsewhere(Govender et al., 2020). For all experiments, a negative 4 mmHg interstitial pressure was applied either after baseline or 5 minutes after 40% hemorrhage, protocol detailed below. The degree of negative pressure applied was manually adjusted throughout the entire experiment to account for air leak.

### 2.4 Hemorrhage and negative pressure protocol

**FIGURE 2:**
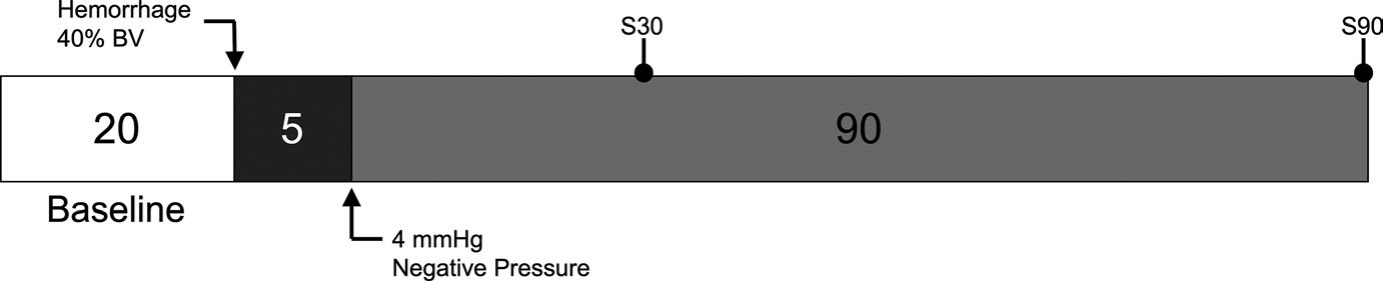
Experimental Timeline. Acute hemorrhage was produced by withdrawal of 40% of the total animal blood volume (BV) via the carotid artery catheter within 5 minutes. BV was estimated as 7% of animal body weight. The parameters analyzed were measured before hemorrhage induction (baseline), and 30 and 90 minutes after hemorrhage.

Acute hemorrhage was produced by withdrawal of 40% of the total animal blood volume (BV) via the carotid artery catheter within 5 minutes. BV was estimated as 7% of animal body weight. The parameters analyzed were measured before hemorrhage induction (baseline), and 30 and 90 minutes after hemorrhage. A schematic timeline of the protocol is shown in Figure 2.

### 2.5 Systemic hemodynamic and blood gas parameter

Mean arterial pressure (MAP) and heart rate (HR) were continuously recorded throughout the entire experiment (MP150; Biopac Systems, Santa Barbara, CA). Arterial blood was collected in heparinized capillary tubes and analyzed for PaO_2,_ PaCO_2_, anion gap, and pH (Blood Chemistry Analyzer 248; Bayer, Norwood, MA). The Hct was measured from centrifuged arterial blood taken in heparinized capillary tubes. The Hb concentrations were measured spectrophotometrically using the B‐Hemoglobin (HemoCue, Stockholm, Sweden) from a drop of arterial blood(Jahr et al., 2002; Rosenblit et al., 1999).

### 2.6 Microvascular experimental setup

The awake animals were placed in a restraint tube with a slit to accommodate the window chamber. The restraint tube was then fixed to a microscopic stage of a transillumination intravital microscope (BX51WI; Olympus, New Hyde Park, NY). Measurements were performed after 20 minutes to allow for adjustment to the tube environment with a 40× (LUMPFL‐WIR, numerical aperture of 0.8; Olympus) water immersion objective.

### 2.7 Microhemodynamics

Detailed mappings of the chamber vasculature were acquired at baseline to ensure the same vessels were followed throughout the experiment. Arteriolar and venular blood flow velocities were measured online using the photodiode cross‐correlation method (Photo‐Diode/Velocity; Vista Electronics, San Diego, CA)(Villela et al., 2011). The measured centerline velocity (V) was normalized to baseline blood vessel measurements to obtain the normalized mean RBC velocity. A video image shearing method was used to measure blood vessel diameter (D), which was normalized to the baseline recording. Blood flow (Q) was computed using Poiseuille's law, Q = π x V x (D/2)^2^ and normalized to the flow computed at baseline.

### 2.8 Functional capillary density

Functional capillaries were defined as capillary segments with transit of at least a single RBC in a 15‐second interval. FCD was assessed in 10 successive microscopic fields, totaling a region of 0.46 mm^2^. The fields were chosen at baseline by a distinctive anatomical (i.e., large bifurcation) or structural landmark. Observation of the fields was performed systematically by adjusting the microscope field of view by one field width in 10 successive steps, each 240 μ m in length relative to the tissue. Each field had between 2 and 10 capillary segments with RBC flow at baseline. FCD is measured in cm^−1^ and calculated by adding the length of capillaries that had RBC transit in the field of view and dividing over the area of the microscope field of view. The relative change in FCD from baseline after each intervention is indicative of microvascular tissue perfusion(Nolte et al., 1995).

### 2.9 Statistical Analysis

All data are represented as mean ±SD. Data were compared using a two‐way ANOVA, with row and column factors being time after treatment and group, respectively. When appropriate, post hoc analysis was performed with Holm–Sidak multiple comparisons test for parametric data. Microhemodynamic data are presented as ratios relative to baseline to account for differences in vessel diameter. The same blood vessels and capillary fields were monitored throughout each experiment to allow for direct comparisons to baseline. Comparisons to baseline were via Student's t test. All statistical analyses were performed in GraphPad Prism 8.3 (GraphPad Software, San Diego, CA). Changes were considered significant if p values were <0.05.

## 3 RESULTS

A total of fifteen animals (n = 15) were included in this study. Animals were randomly assigned to each experimental group based on application of negative pressure. These groups were (i) negative pressure during normovolemia, labeled Negative Pressure (no Hemorrhage), n = 5; (ii) hypovolemia (40% BV hemorrhage) without application of negative pressure, labeled 40% Hemorrhage, n = 5; and (iii) negative pressure during hypovolemia (40% BV hemorrhage), labeled Negative Pressure after 40% Hemorrhage, n = 5. For each animal, between four and six arterioles and four and six venules were selected after application of systemic and vessel inclusion criteria. The parameters analyzed were measured before hemorrhage induction (baseline), and 30 and 90 minutes after hemorrhage. There were no significant differences in hematocrit, systemic, or microhemodynamic parameters at baseline among all animals in all groups.

### 3.1 Systemic Parameters

**FIGURE 3:**
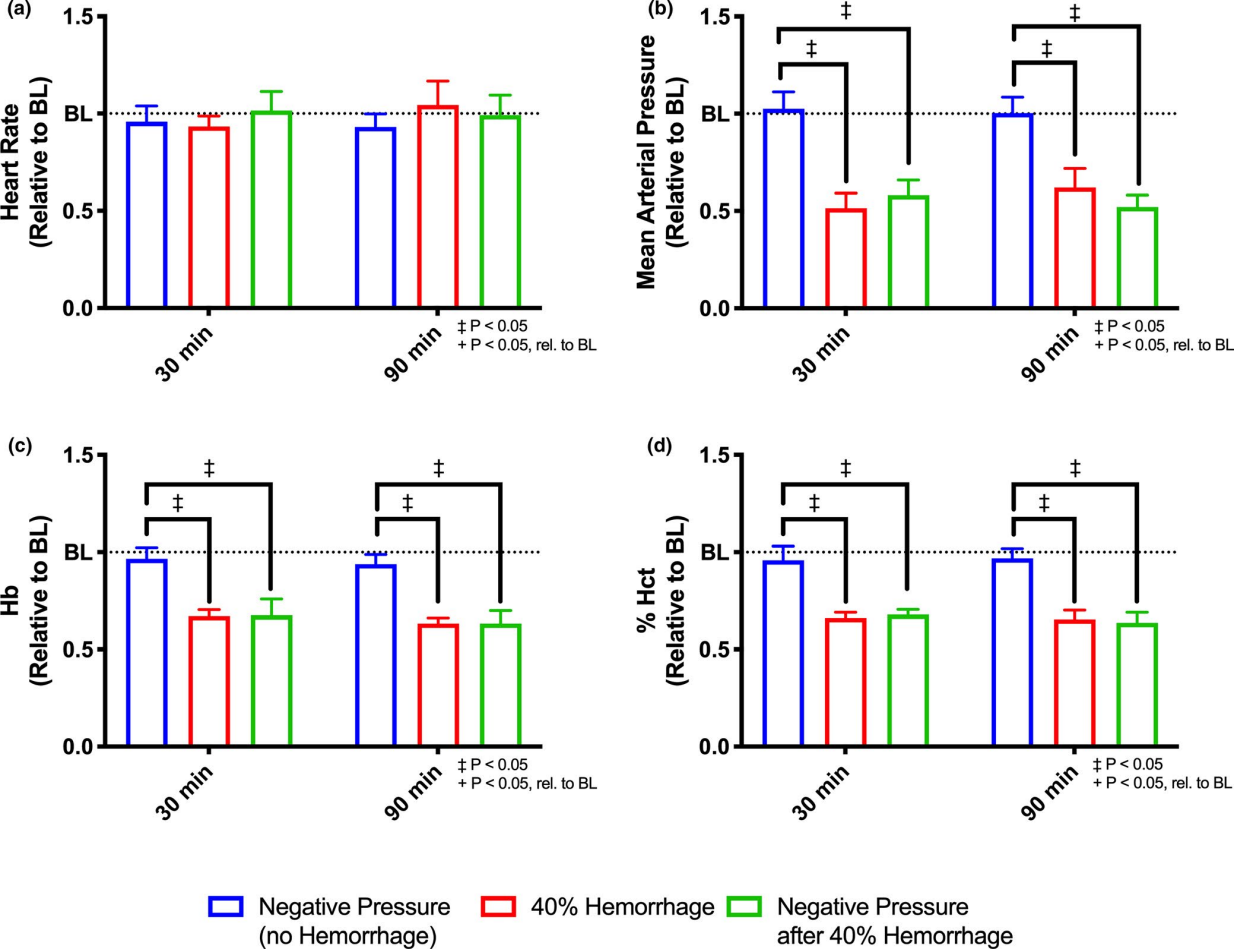
Systemic Hemodynamics. Systemic hemodynamic parameters were assessed at baseline, 30 minutes after intervention, and 90 minutes after intervention for all groups, (i) negative pressure during normovolemia, labeled Negative Pressure (no Hemorrhage), n = 5; (ii) hypovolemia (40% BV hemorrhage) without application of negative pressure, labeled 40% Hemorrhage, n = 5; and (iii) negative pressure during hypovolemia (40% BV hemorrhage), labeled Negative Pressure after 40% Hemorrhage, n = 5. Hemodynamics shown include the following: (a) heart rate, (b) mean arterial pressure, (c) hemoglobin (Hb) concentration (g/dL), and (d) % hematocrit (Hct). All results were normalized relative to measured results at baseline. Changes in MAP, Hb, and % Hct reflect the expected changes. +: *p* < 0.05 rel. to baseline. ‡:*p* < 0.05 between groups.

Systemic hemodynamic parameters are summarized in Figure 3. Briefly, there were no significant differences in heart rate between groups at 30 and 90 minutes, though the change in heart rate was significant with time (*p* < 0.05). Mean arterial pressure (MAP), hematocrit, and hemoglobin reflect changes expected from 40% BV hemorrhage.

### 3.2 Microvascular Diameter

**FIGURE 4:**
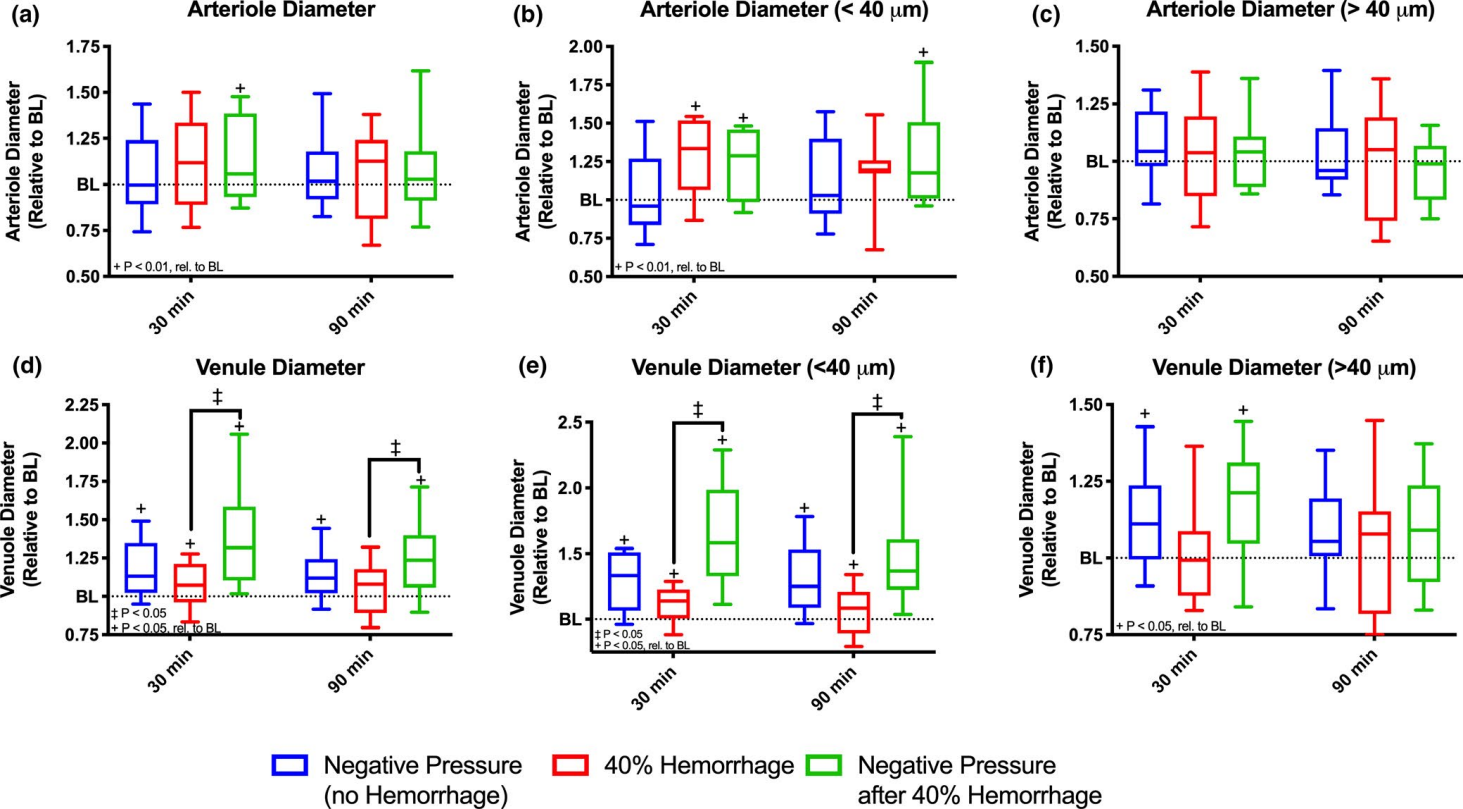
Arteriolar and Venular Diameter with Subgroup Analysis. Arteriolar and venular diameter was measured using an image shearing technique and assessed at baseline, 30 minutes after intervention, and 90 minutes after intervention for all groups, (i) negative pressure during normovolemia, labeled Negative Pressure (no Hemorrhage), n = 5; (ii) hypovolemia (40% BV hemorrhage) without application of negative pressure, labeled 40% Hemorrhage, n = 5; and (iii) negative pressure during hypovolemia (40% BV hemorrhage), labeled Negative Pressure after 40% Hemorrhage, n = 5. All values are normalized with respect to baseline. Subgroup analysis was performed based on vessel size. Plots shown are as follows: (a) arteriolar diameter (All), (b) arteriole diameter (< 40 μm), (c) arteriole diameter (> 40 μm), (d) venular diameter (All), (e) venular diameter (< 40 μm), and (f) venular diameter (> 40 μm). +: *p* < 0.05 rel. to baseline. ‡:*p* < 0.05 between groups.

Arteriolar and venular diameters for all groups are summarized in Figure 4. Application of negative pressure after 40% hemorrhage significantly increased arteriolar diameter at 30 minutes (*p* < 0.05) compared to baseline. However, no significant changes in diameter were observed compared to baseline at 90 minutes in this group (Figure 4a). Subgroup analysis was performed based on arteriolar size. For arterioles <40 μ m, a statistically significant increase (*p* < 0.05) in diameter was observed with 40% hemorrhage both with and without application of negative pressure at 30 minutes. Though, at 90 minutes, this significant increase in diameter was sustained only with application of negative pressure with 40% hemorrhage (Figure 4b). For larger arterioles >40 μ m, no significant differences were observed relative to baseline and between groups for all times assessed (Figure 4c).

There was a significant increase in venular diameter (*p* < 0.05) relative to baseline for all groups at 30 minutes. Similarly, at 90 minutes, venular diameter was significantly increased relative to baseline with application of negative pressure regardless of volume status (Figure 4d). Venular diameter was significantly larger with application of negative pressure after 40% hemorrhage compared to hemorrhage at all time points (Figure 4d). Similar results were observed when analyzing only venules <40 μ m (Figure 4e). For venules >40 μ m, a significant increase in diameter was observed relative to baseline with application of negative pressure regardless of volume status at 30 minutes (Figure 4f).

### 3.3 Microvascular Velocity

**FIGURE 5:**
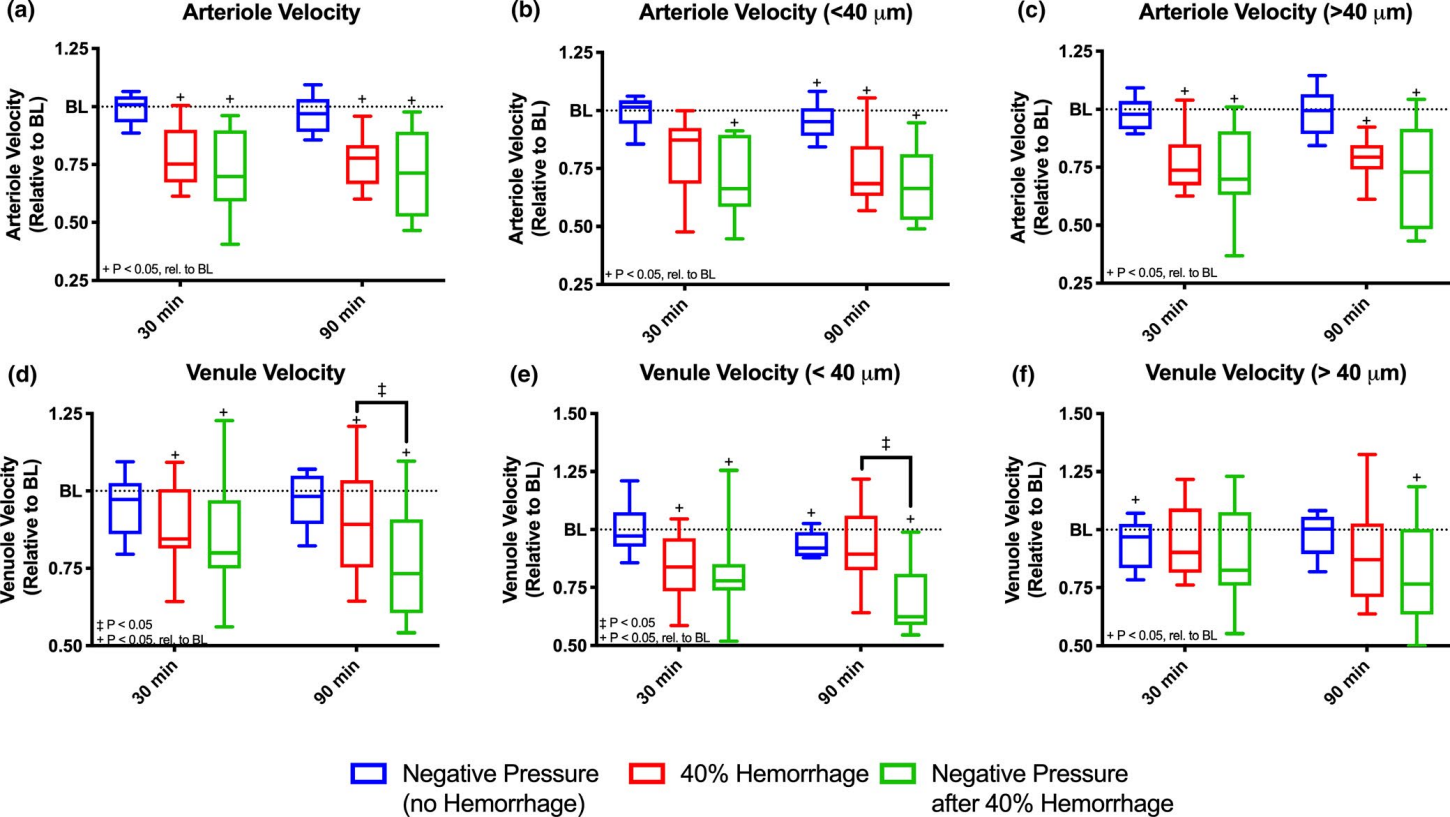
Arteriolar and Venular Velocity with Subgroup Analysis. Arteriolar and venular velocities were measured using intravital microscopy and assessed at baseline, 30 minutes after intervention, and 90 minutes after intervention for all groups, (i) negative pressure during normovolemia, labeled Negative Pressure (no Hemorrhage), n = 5; (ii) hypovolemia (40% BV hemorrhage) without application of negative pressure, labeled 40% Hemorrhage, n = 5; and (iii) negative pressure during hypovolemia (40% BV hemorrhage), labeled Negative Pressure after 40% Hemorrhage, n = 5. All values are normalized with respect to baseline. Subgroup analysis was performed based on vessel size. Plots shown are as follows: (a) arteriolar velocity (All), (b) arteriole velocity (< 40 μm), (c) arteriole velocity (> 40 μm), (d) venular velocity (All), (e) venular velocity (< 40 μm), and (f) venular velocity (> 40 μm). +: *p* < 0.05 rel. to baseline. ‡:*p* < 0.05 between groups.

Arteriolar and venular velocities in all groups are summarized in Figure 5. A significant decrease in velocity (*p* < 0.05) was observed in arterioles after 40% hemorrhage regardless of negative pressure application relative to baseline at all time points (Figure 5a). For arterioles <40 μ m, a statistically significant decrease (*p* < 0.05) in velocity was observed relative to baseline after 40% hemorrhage with application of negative pressure at 30 minutes. At 90 minutes, small (<40 μ m) arteriolar velocity was decreased relative to baseline for all groups (Figure 5b). For arterioles >40 μ m, velocity was significantly decreased with 40% hemorrhage regardless of application of negative pressure at all time points (Figure 5c).

Venular velocity was significantly decreased relative to baseline after 40% hemorrhage regardless of application of negative pressure at all time points. Additionally, after 90 minutes venular velocity was significantly decreased after application of negative pressure after 40% hemorrhage compared to hemorrhage alone (Figure 5d). Subgroup analysis was performed based on venular size. Briefly, results were similar to those described above for venules <40 μ m; however, a significant decrease in velocity was observed relative to baseline with application of negative pressure during normovolemia at 90 minutes, though not in 40% hemorrhage alone (Figure 5e). In venules >40 μ m, a statistically significant decrease in venular velocity was observed relative to baseline only in the Negative Pressure (no Hemorrhage) group at 30 minutes, though not at 90 minutes. Surprisingly, application of negative pressure after 40% hemorrhage resulted in a significant decrease in venular velocity relative to baseline (Figure 5f).

### 3.4 Microvascular Flow

**FIGURE 6:**
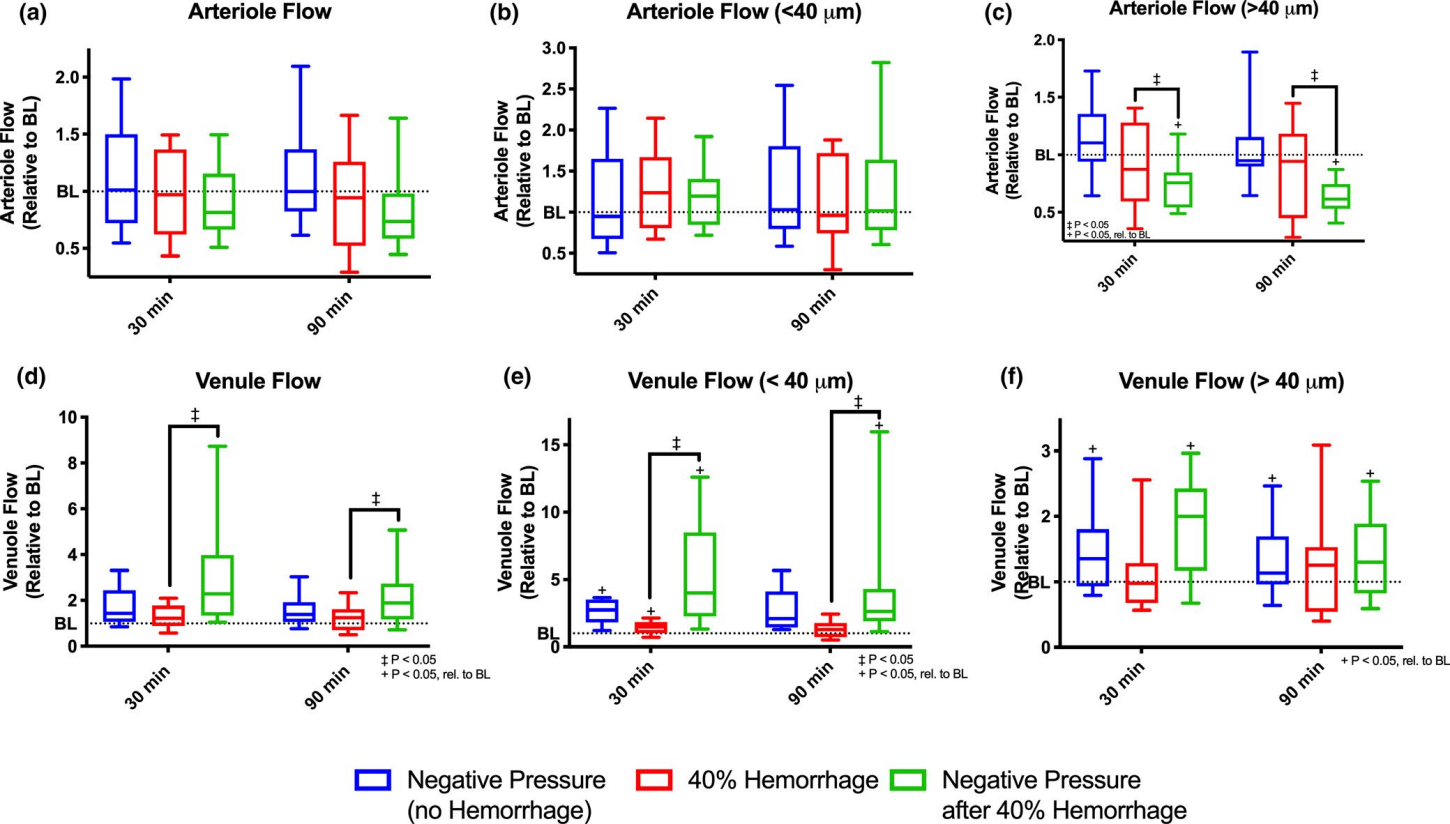
Arteriolar and Venular Flow with Subgroup Analysis. Arteriolar flow and venular flow were calculated using Poiseuille's law and assessed at baseline, 30 minutes after intervention, and 90 minutes after intervention for all groups, (i) negative pressure during normovolemia, labeled Negative Pressure (no Hemorrhage), n = 5; (ii) hypovolemia (40% BV hemorrhage) without application of negative pressure, labeled 40% Hemorrhage, n = 5; and (iii) negative pressure during hypovolemia (40% BV hemorrhage), labeled Negative Pressure after 40% Hemorrhage, n = 5. All values are normalized with respect to baseline. Subgroup analysis was performed based on vessel size. Plots shown are as follows: (a) arteriolar flow (All), (b) arteriole flow (< 40 μm), (c) arteriole flow (> 40 μm), (d) venular flow (All), (e) venular flow (< 40 μm), and (f) venular flow (> 40 μm). +: *p* < 0.05 rel. to baseline. ‡:*p* < 0.05 between groups.

Flow was calculated for both arterioles and venules from Poiseuille's equation with the measured diameters and velocities described above (Figure 6). No statistically significant differences in arteriolar flow relative to baseline or between groups were observed (Figure 6a). Subgroup analysis revealed similar results for arteriolar flow for small arterioles <40 μ m (Figure 6b). In large arterioles (>40 μ m), a statistically significant decrease in flow relative to baseline was observed in the Negative Pressure after 40% Hemorrhage group at all time points (Figure 6c). Furthermore, arteriolar flow was significantly decreased with application of negative pressure with the 40% hemorrhage compared to hemorrhage alone.

A significant increase in venular flow was observed with application of negative pressure with 40% hemorrhage compared to hemorrhage alone at all time points (Figure 6d). Subgroup analysis revealed similar results for arteriolar flow for small venules <40 μ m (Figure 6e). In large venules (>40 μ m), a statistically significant increase in flow was observed relative to baseline with application of negative pressure regardless of volume status (Figure 6f). No statistically significant changes between groups were observed for these venules.

### 3.5 Functional Capillary Density

**FIGURE 7:**
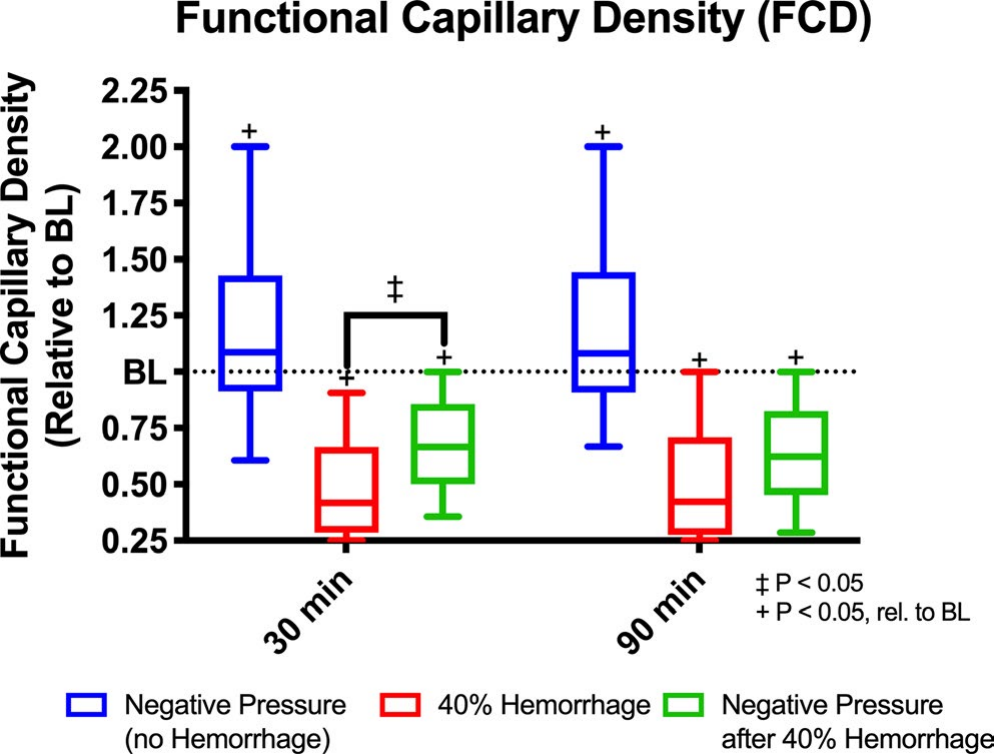
Functional Capillary Density. Functional capillary density was assessed using intravital microscopy at baseline, 30 minutes after intervention, and 90 minutes after intervention for all groups, (i) negative pressure during normovolemia, labeled Negative Pressure (no Hemorrhage), n = 5; (ii) hypovolemia (40% BV hemorrhage) without application of negative pressure, labeled 40% Hemorrhage, n = 5; and (iii) negative pressure during hypovolemia (40% BV hemorrhage), labeled Negative Pressure after 40% Hemorrhage, n = 5. +: *p* < 0.05 rel. to baseline. ‡:*p* < 0.05 between groups.

Functional capillary density (FCD) for all groups is shown in Figure 7. A statistically significant increase in FCD was observed relative to baseline with application of negative pressure. As expected, a statistically significant decrease in FCD was observed relative to baseline after 40% hemorrhage regardless of volume status. Surprisingly, at 30 minutes, FCD was significantly higher with application of negative pressure after 40% hemorrhage compared to hemorrhage alone (*p* < 0.05). This difference was not sustained 90 minutes after 40% hemorrhage.

## 4 DISCUSSION

This study evaluates the effects of applied negative tissue interstitial pressure on microvascular perfusion both during normovolemia and after hemorrhagic shock. The principle findings of this study are as follows: (i) In normovolemia, application of constant negative tissue interstitial pressure acutely increases FCD and venular outflow, indicating increased microvascular perfusion and intramural capillary pressure, respectively; (ii) in hemorrhagic shock, application of negative pressure acutely improves FCD, though does not normalize it; and (iii) in both normovolemia and hypovolemia, application of negative tissue interstitial pressure increases mean capillary perfusion pressure by increasing venular outflow while maintaining arteriolar inflow. These results suggest that by increasing the hydrostatic pressure gradient between the microvasculature and interstitium, microvascular perfusion can be transiently restored in the absence of volume resuscitation. Importantly, these changes were not sustained in our studies, and alternative interventions may be required for chronic management of hypovolemic shock. Multiple interventions, namely high viscosity plasma expanders, have demonstrated improvement in microvascular perfusion without packed red cell transfusion(Cabrales et al., 2004; Tsai et al., 1998). However, few studies have investigated transfusion‐independent methodologies for restoration of microvascular perfusion in hemorrhagic shock. The findings from this study have significant clinical implications, particularly for reducing edema during surgical wound closure (i.e., negative pressure wound therapy)(Banwell & Musgrave, 2004).

Application of negative pressure did little to mitigate the vascular response in smaller arteriole beds during hemorrhagic shock. Surprisingly, however, there was a significant decrease in flow in larger arterioles that feed these smaller microvascular networks. During hemorrhagic shock, there is a systemic decrease in MAP and microvascular wall shear stress, resulting in activation of the carotid body and local endothelin‐mediated vasoconstriction secondary to transient hypoxia, respectively. A catecholamine surge from carotid body activation results in sympathetic stimulation of vascular α_1_ adrenergic receptors resulting in systemic vasoconstriction. However, it is well known that variability in perfusion pressure and microvascular shear stress results in a heterogeneous myogenic response across any given microvascular network(Kerger et al., 1996, 1999; Meßmer et al., 1973). Changes in vessel diameter and flow therefore depend on the selection of the tissue analyzed and specific vessels measured. In our study, we observed a significant increase in the diameter of small arterioles during hemorrhagic shock in response to decreased perfusion pressure to maintain microvascular flow; this was limited to smaller arterioles as the mechanical properties of larger arterioles limit their maximum diameter(Bohlen and Lash, 1994;23(6_pt_^1^):^757^–^764^.; Lash & Bohlen, 1991; Lash et al., 1991).

The objective of myogenic response is to maintain constant circumferential, or hoop, stress during changes in vascular hydrostatic pressure. This response is responsible for limited changes in flow during hypovolemic shock(Gonzalez‐Fernandez & Ermentrout, 1994; Harder, 1987; Harder et al., 1987; Liu et al., 1994). In our study, application of negative pressure during normovolemia had few effects on arteriolar flow. Surprisingly, application of negative pressure after hemorrhagic shock exacerbated the decrease in arteriolar flow. In this case, the increase in transmural pressure is likely due to the decrease in interstitial pressure being greater than the decrease in capillary pressure.

As discussed, application of constant negative tissue interstitial pressure acutely increases venular outflow both during normovolemia and hypovolemia in vessels <40 μ m. These changes are due to the increased diameter of venules and are reflective of their passive mechanical properties. These changes are likely responsible for the transient increase in FCD with application of negative pressure during normovolemia, as FCD is linearly proportional to capillary transmural pressure(Cabrales et al., 2004). Furthermore, the increase in capillary transmural pressure in response to negative pressure was significant enough to improve FCD transiently after hemorrhage, though not entirely corrective.

FCD and capillary inflow are grossly dependent on two factors, precapillary microvascular resistance and pericyte activity at the capillary orifice(Honig et al., 1980; Tyml & Groom, 1980). Both these factors are responsible for adjusting capillary transmural pressure, which is linearly proportional to perfusion(Cabrales et al., 2004; Honig et al., 1982). Surprisingly, despite the decrease in precapillary flow in our results, we observed an increase in FCD. These data suggest that altering tissue hydrostatic pressure and thus Starling Forces alters the function of pericytes, the other component responsible for maintaining FCD. Future studies should aim to investigate the specific molecular response to negative tissue interstitial pressure in pericytes.

The clinical relevance of exogenous application of negative tissue interstitial pressure has primarily been limited to negative pressure wound therapy (NPWT) during surgical wound closure in orthopedic surgery and diabetes(Banwell & Musgrave, 2004; Dumville et al., 2013;(10).; Huang et al., 2014; Robert, 2017). However, in these situations, the objective of NPWT is to decrease interstitial edema to allow for more effective wound remodeling. Studies to assess the effects of NPWT on local microvascular perfusion and oxygenation have been limited, though results have been conflicting(Borgquist et al., 2010; Glass et al., 2014; Ma et al., 2017; Muenchow et al., 2019; Sogorski et al., 2018). Sogorski et al. demonstrated a significant increase in oxygen saturation and red cell velocity in the microcirculation(Sogorski et al., 2018). However, at low magnitudes of negative pressure in pigs, Borgquist et al. demonstrated a significant decrease in vascular blood flow, consistent with the arteriolar findings observed in our studies(Borgquist et al., 2010). Most studies, however, assessed total tissue perfusion and did not assess differences in arteriolar and venous flow and capillary perfusion. Furthermore, these studies investigated pressures in the range of −100 mmHg, in which vascular compression is a significant factor. Surprisingly, molecular studies have demonstrated accelerated microvessel maturation and increased pericyte recruitment with application of negative pressure by upregulation of angiogenin 1, tyrosine kinase receptor‐2, a‐smooth muscle actin, and collagen type IV(Ma et al., 2017). Future studies are needed to further elucidate the mechanisms of pericyte function with exogenous negative pressure, especially in the context of hemorrhagic shock.

Our study has several limitations. Only animals that survived the shock protocol were included in the analysis. Kerger et al. demonstrated that arteriolar flow, vasomotion, and FCD are significantly different in animals that survived hemorrhagic shock compared to those that do not(Kerger et al., 1996). Future studies are required to assess the effect of negative pressure on survival in this protocol. Microvascular flow and FCD are dependent on vessel selection, which contributes to the high variability observed. Due to technical limitations, capillary transmural pressure was not directly measured, and hypotheses about its relevance for the results observed in this study must be considered with caution. Furthermore, the tissue interstitial pressure was not directly measured in this study, so it is unknown whether application was homogeneous within the entire chamber field of view, which may alter observed microvascular hemodynamics.

## 5 CONCLUSION

In summary, this study evaluates the effects of application negative tissue interstitial pressure on microvascular perfusion both during normovolemia and after hemorrhagic shock. Our results demonstrate that application of negative pressure acutely improves FCD after hemorrhagic shock, though it does not normalize it. These results suggest that by increasing the hydrostatic pressure gradient between the microvasculature and interstitium, microvascular perfusion can be transiently restored in the absence of volume resuscitation. The physiology from this study has significant clinical implications, particularly in NPWT and offers an alternative mechanism to improve microvascular perfusion during hypovolemic shock. Future studies are required to assess the role of pericyte function and calcium transience after application of negative tissue interstitial pressure in these situations.

## CONFLICT OF INTEREST

There is no potential conflict of interest, real or perceived, by the authors.

## AUTHOR CONTRIBUTIONS

VJ, VJ, and PC conceived and designed the study. VJ and VJ performed experiments and collected measurements. VJ, CM, and PC performed statistical analysis. VJ and PC wrote the manuscript. VJ, CM, and KG provided additional assistance drafting the manuscript. VJ and AT designed the chamber used in the experiments. All authors revised the manuscript critically for important intellectual content, and all authors read and approved the final version to be published.
